# Supplementary material for: A multicenter prospective study to determine the optimal range of lymph node dissection in pancreatic cancer surgery after neoadjuvant chemotherapy (LYMRIN-Trial): Project study by the Japan Pancreas Society and JON 2302-P
Source: PLoS One. 2025 Jun 17;20(6):e0325667. doi: 10.1371/journal.pone.0325667 (PMC12173190; doi:10.1371/journal.pone.0325667)
Supplement: S1 Table — Station numbers and names of lymph nodes related to the pancreas in this study. (DOCX) [file pone.0325667.s001.docx]

**Supporting table 1. Station numbers and names of lymph nodes related to the pancreas.**

| **Number** | **Name** |
| --- | --- |
| 1 | Right cardial lymph nodes |
| 2 | Left cardial lymph nodes |
| 3 | Lymph nodes along the lesser curvature of the stomach |
| 4 | Lymph nodes along the greater curvature of the stomach |
| 5 | Suprapyloric lymph nodes |
| 6 | Infrapyloric lymph nodes |
| 7 | Lymph nodes along left gastric artery |
| 8a | Lymph nodes in the anterosuperior group along common hepatic artery |
| 8p | Lymph nodes in the posterior group along common hepatic artery |
| 9 | Lymph nodes around celiac artery |
| 10 | Lymph nodes at the splenic hilum |
| 11p | Lymph nodes along the proximal splenic artery |
| 11d | Lymph nodes along the distal splenic artery |
| 12a | Lymph nodes along the hepatic artery |
| 12p | Lymph nodes along portal vein |
| 12b | Lymph nodes along the bile duct |
| 13 | Lymph nodes on the posterior aspect of the head of the pancreas |
| 14t | Lymph nodes along the superior mesenteric artery; tumor side |
| 14op | Lymph nodes along the superior mesenteric artery; opposite side of tumor |
| 15 | Lymph nodes along middle colic artery |
| 16a | Lymph nodes around the abdominal aorta a |
| 16b | Lymph nodes around the abdominal aorta b |
| 17 | Lymph nodes on the anterior surface of the head of the pancreas |
| 18 | Lymph nodes along the inferior margin of the pancreas |
