# Supplementary material for: A multicenter prospective study to determine the optimal range of lymph node dissection in pancreatic cancer surgery after neoadjuvant chemotherapy (LYMRIN-Trial): Project study by the Japan Pancreas Society and JON 2302-P
Source: PLoS One. 2025 Jun 17;20(6):e0325667. doi: 10.1371/journal.pone.0325667 (PMC12173190; doi:10.1371/journal.pone.0325667)
Supplement: S1 File — List of facilities participating in this study. (DOCX) [file pone.0325667.s002.docx]

**Supporting file 1.** Institution list

| Asahikawa Medical University |
| --- |
| Chiba Cancer Center |
| Chiba University |
| Dokkyo Medical University |
| Dokkyo Medical University Saitama Medical Center |
| Fujita Health University |
| Fujita Health University Bantane Hospital |
| Hirosaki University |
| Hiroshima University |
| Hokkaido University |
| Hyogo Medical University |
| JA Onomichi General Hospital |
| Jichi Medical University |
| Jichi Medical University Saitama Medical Center |
| Juntendo University |
| Kagawa University |
| Kagoshima University |
| Kanagawa Cancer Center |
| Kansai Medical University |
| Keio University |
| Kindai University |
| Kyorin University |
| Kyoto University |
| Kyusyu University |
| Mie University |
| Miyazaki University |
| Nagoya Central Hospital |
| Nagoya University |
| Nara Medical University |
| Ohara HealthCare Foundation, Kurashiki Central Hospital |
| Osaka City General Hospital |
| Osaka International Cancer Institute |
| Osaka University |
| Saitama Cancer Center |
| Sapporo Medical University |
| Showa University |
| Tohoku University |
| Tokyo Medical University |
| University of Toyama |
| Wakayama Medical University |
| Yamagata University |
| Yamaguchi University |
