# Supplementary material for: A multicenter prospective study to determine the optimal range of lymph node dissection in pancreatic cancer surgery after neoadjuvant chemotherapy (LYMRIN-Trial): Project study by the Japan Pancreas Society and JON 2302-P
Source: PLoS One. 2025 Jun 17;20(6):e0325667. doi: 10.1371/journal.pone.0325667 (PMC12173190; doi:10.1371/journal.pone.0325667)
Supplement: S1 Protocol — Study protocol of LYMRIN trial. (DOCX) [file pone.0325667.s003.docx]

Clinical Study Protocol

A multicenter prospective study to determine the optimal range of

lymph node dissection in pancreatic cancer surgery after neoadjuvant chemotherapy

-Project study by the Japan Pancreas Society and JON 2302-P

Protocol version

Version 1.0 30^th^, October 2023

Version 1.1 12^th^, January 2024

Version 1.2 6^th^ , March 2024

Version 1.3 19^th^, July 2024

Table of Contents

[0．Summary 2](#_Toc146659914)

[1．Title 8](#_Toc146659915)

[2．Implementation system 8](#_Toc146659916)

[3．Background](#_Toc146659917) 8

[4．Objective](#_Toc146659918) 9

[5．Basis of scientific rationality and significance](#_Toc146659919) 9

[6．Inclusion criteria/ Exclusion criteria](#_Toc146659920) 9

[7．Methods and period 1](#_Toc146659921)1

[8．Evaluation, clinical examination, schedule 2](#_Toc146659922)4

[9．Informed consent 2](#_Toc146659923)7

[10．Methods of handling, storing and disposing of personal information 2](#_Toc146659924)8

[11．Comprehensive assessment of the burden. Risks and benefit to the research patients 2](#_Toc146659925)9

[12．Funding and conflicts of interest 3](#_Toc146659926)1

[13．Response to adverse events 3](#_Toc146659927)1

[14．Monitoring 3](#_Toc146659928)1

[References 3](#_Toc146659936)3

[APPENDIX　1 3](#_Toc146659937)5

# **0．Summary**

**0.1　Title**

A multicenter prospective study to determine the optimal range of lymph node dissection in pancreatic cancer surgery after neoadjuvant chemotherapy -Project study by the Japan Pancreas Society and JON 2302-P-


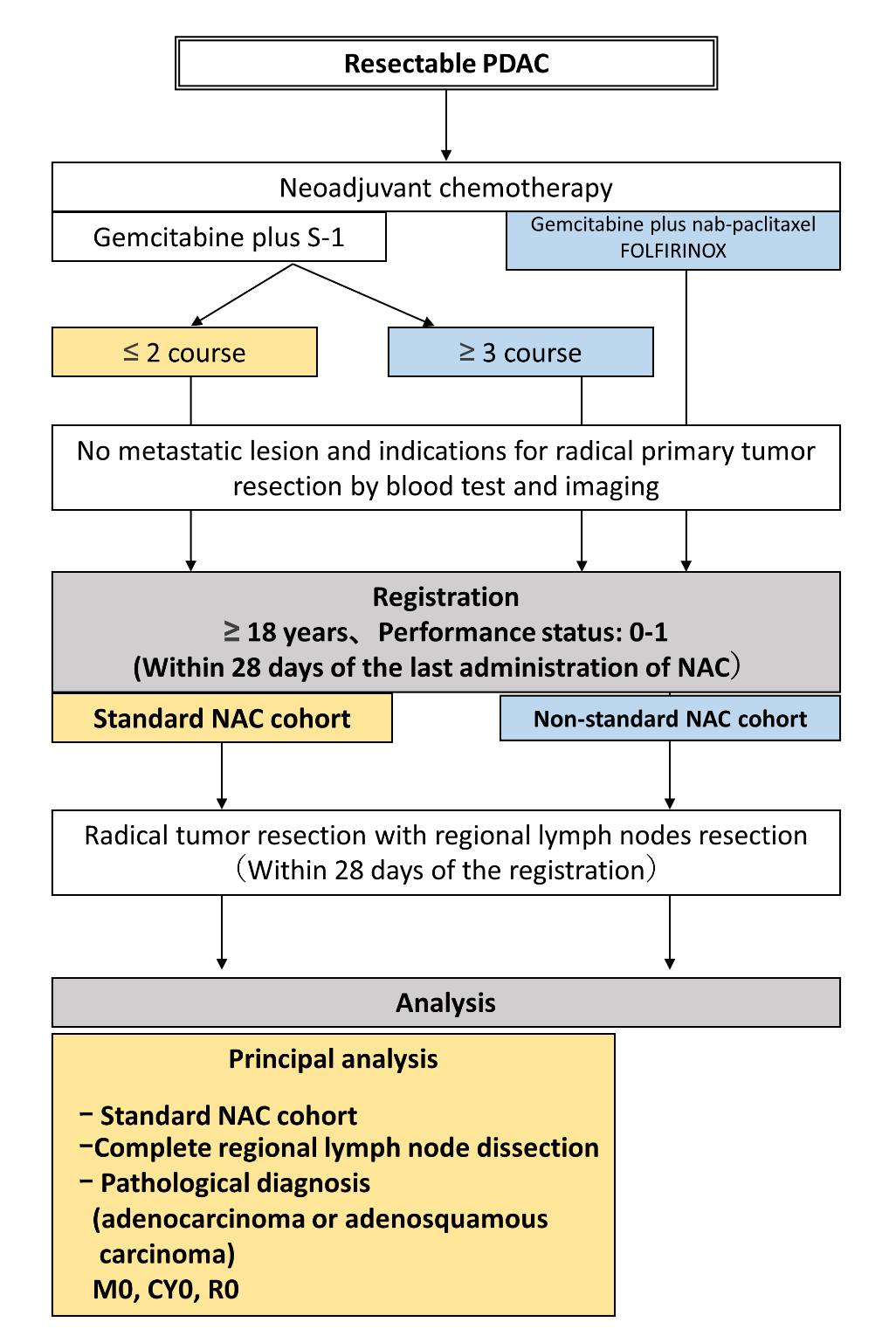
**0.2 Flow chart**

* For postoperative adjuvant therapy, 6 months of S-1 therapy is recommended.

However, the timing of initiation, treatment and types of postoperative adjuvant therapy are not limited.

**0.3 Aim and endpoints**

The aim of the LYMRIN trial is to determine the optimal range of lymph node dissection for patients with resectable pancreatic cancer after neoadjuvant chemotherapy (NAC). This will be achieved by clarifying the index of estimated benefit of lymph node dissection, which will be calculated by the metastasis rate of each lymph node station and the survival rate of the patient with lymph node metastasis.

Primary endpoint

This clinical trial will primarily evaluate the metastatic rate of each lymph node station in a standard cohort treated with NAC.

Secondary endpoints

The secondary endpoints include the index of estimated benefit of each lymph node dissection (metastatic rate x 3- or 5-year survival), disease-free survival, overall survival, cancer-specific survival, postoperative complications, the location of the relapse site in the standard and nonstandard cohorts, and the metastatic rate of each lymph node station in the nonstandard cohort. The results will be published in a conference or in a paper.

**0.4　Eligible patients**

Patients who meet all the following inclusion criteria and none of the exclusion criteria will be considered eligible for enrollment (eligible patients).

## 0.4.1．Inclusion criteria

1) Diagnosis：one of the following ① to ③ is fulfilled.

①　Histological diagnosis as PDAC (adenocarcinoma, adenosquamous carcinoma)

②　Cytological diagnosis as PDAC (Class IV or Class V)

③　Clinically diagnosed as PDAC from contrast-enhanced CT or MRI, although no pathological diagnosis has been made.

2) The primary tumor is diagnosed as resectable

3) No history of upper abdominal surgery

4) Surgical resection with regional lymph nodes (Table 2) dissection is possible with imaging

5) Peritoneal washing cytology: negative (if applicable)

6) Neoadjuvant chemotherapy was performed

7) Tumors determined to be suitable for radical primary resection within 28 days after neoadjuvant chemotherapy

8) Planned pancreatoduodenectomy for pancreatic head cancer, distal pancreatectomy for pancreatic body-tail cancer

9) Age: 18 years or older

10) Performance status (Eastern Cooperative Oncology Group scale): 0-1

11) Ability to understand and willingness to sign written informed consent document

0.4.2．Exclusion criteria

1) Active multiple primary cancers (synchronous or asynchronous within 5 years). However, a history of cancer equivalent to a 5-year relative survival rate of 95% or more, such as stage I prostate cancer, stage 0/I laryngeal cancer with a complete response to radiotherapy, or cancer of the following pathological stages that has been completely resected, even if disease-free for less than 5 years, is not included in active multiple/polycystic cancers.

Gastric cancer : Stage 0 -I, Colon cancer : Stage 0-I, Rectal cancer : Stage 0 -I, Esophageal cancer : Stage 0、Breast cancer (non-invasive) : Stage 0, Breast cancer (invasive, Paget) : Stage 0 -IIA, Uterine body cancer : Stage I, Prostate cancer : Stage I -II, Cervical cancer: Stage 0, Thyroid cancer: Stage I-III, Renal cancer: Stage I

2) Serious comorbidities (heart failure, interstitial pneumonia, renal failure, liver failure, intestinal paralysis, intestinal obstruction, poorly controlled diabetes, poorly controlled hypertension, etc.)

3) Protocol treatment (radical primary tumor resection and regional lymph node dissection) cannot be safely performed

4) Receiving pre-treatment (radiotherapy, immunotherapy, etc.) other than neoadjuvant chemotherapy

5) Pregnant or lactating women and women of childbearing potential

6) Severe psychological or neurological disease

**0.5 Registration**

Patients who are scheduled to receive the standard neoadjuvant chemotherapy “gemcitabine + S-1 combination therapy” (“GS therapy”) will be enrolled in the standard NAC cohort if they have completed 2 courses or less of NAC GS therapy, undergo diagnostic imaging and blood tests to evaluate efficacy, and are judged to be eligible for radical primary tumor resection without new lesions or distant metastases. Registration is required within 28 days of the last dose of NAC.

Patients who received other preoperative chemotherapy (gemcitabine plus nab-paclitaxel, FOLFIRINOX (original or variant), or 3 or more courses of GS therapy) will be enrolled as a nonstandard cohort after similar evaluation.

**0.6 Treatment protocol**

Treatment protocol in this study is defined as radical primary pancreatectomy (pancreaticoduodenectomy or distal pancreatectomy) and regional lymph node dissection from the start to the end of surgery.

0.6.1. Primary tumor resection

Primary tumor resection is performed within 28 days of registration. Surgical resection in this study allows open, laparoscopic and robot-assisted pancreatectomy.

・Pancreatic head cancer：pancreatoduodenectomy

・Pancreatic body cancer：distal pancreatectomy

・Pancreatic tail cancer：distal pancreatectomy

Pancreatoduodenectomy for pancreatic body cancer is not acceptable and included in the analysis as a deviation of treatment protocol. Patients who underwent total pancreatectomy or major artery resection (celiac artery, hepatic artery, variant hepatic artery, superior mesenteric artery) are similarly not included in the analysis.

0.6.2.　Regional lymph nodes dissection

Regional lymph nodes by tumor location

| Tumor location of pancreas | Regional lymph nodes |
| --- | --- |
| Head | 6, 8a, 8p, 12a, 12b, 12p, 13, 14t,14op, 17 |
| Body | 8a, 8p, 9, 10, 11p, 11d, 14t, 18 |
| Tail | 8a. 9, 10, 11p, 11d, 18 |


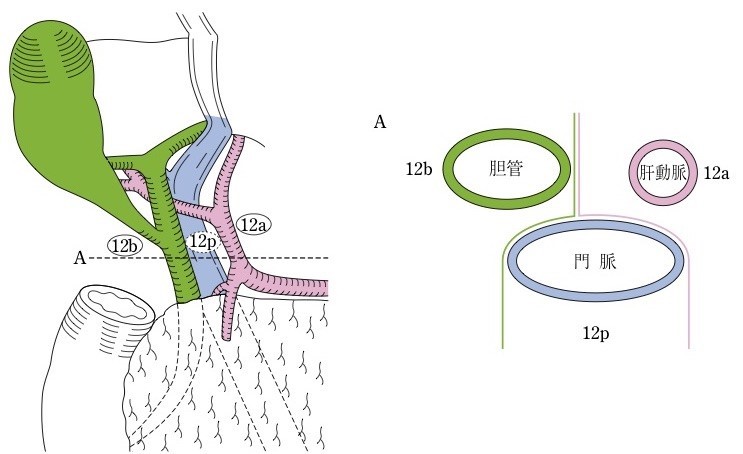


HA

CBD

PV

PV

Figure 3．Hepatoduodenal ligament and lymph nodes


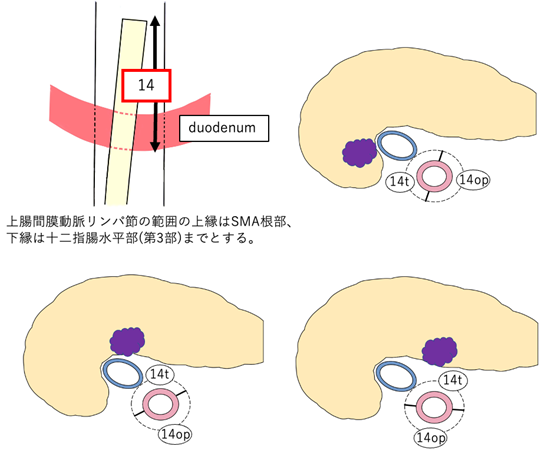


Figure 4．Superior mesenteric artery and lymph nodes(1)

**0.7 　Statistical analysis**

0.7.1.　 Principal analysis requirements

1. Radical resection within 28 days of registration
2. Pancreatectomy with regional lymph nodes dissection shown in Table 1.
3. Pathological examination diagnoses invasive ductal adenocarcinoma (adenocarcinoma, adenosquamous carcinoma). R0 resection is required. R0 is defined as the absence of cancer cells within 1mm of the edge by Royal College of Pathology(2).
4. Abdominal cytology negative.
5. No distant metastasis.
6. Not all of the following
7. Major artery resection and reconstruction
8. Surgical mortality

0.7.2. Other analysis

In the non-standard cohort patients and in the standard NAC cohort patients who do not meet the criteria for the main analysis, statistical analysis will be performed as in the main analysis.

**0.8 Sample size estimation**

The expected enrollment for this trial is 545 patients in the standard NAC cohort (200 patients with pancreatic head cancer, 165 patients with pancreatic body cancer, and 180 patients with pancreatic tail cancer). Registration of sites that have reached the planned number of registrations will be considered complete, and only those sites that have not reached the planned number of registrations will continue to be registered.

Enrollment of the non-standard cohort will be terminated when the number of patients meeting the primary analysis criteria in all sites (head, body, tail) is completed in standard NAC cohort.

**0.9 Study periods**

Registration：3 years（the date of approval ～ September 30, 2026）

Follow-up period：5 years after the end of the registration period

Total study period：8 years（the date of approval ～ September 30, 2031）

# 1．Title

A multicenter prospective study to determine the optimal range of lymph node dissection in pancreatic cancer surgery after neoadjuvant chemotherapy -Project study by the Japan Pancreas Society and JON 2302-P-

# 2．Implementation system

Principal investigator：Tsutomu Fujii

Department of Surgery and Science, University of Toyama.

Collaborate institution

Japan Pancreas Society

Japan Oncology Network HPB

Institutions with at least 40 pancreatectomies per year are considered to be study participants.

# 3．Background

Pancreatic ductal adenocarcinoma (PDAC) is one of the most lethal types of gastrointestinal cancers and the seventh leading cause of cancer-related death worldwide(3). The number of deaths due to pancreatic cancer continues to increase, with a 5-year survival rate of only 13%(4). Pancreatic cancer can be classified as resectable, borderline resectable, or unresectable based on whether R0 surgery without gross or histologic evidence of residual cancer is possible by standard surgery(5). According to the results of the Prep-02/JSAP-05 study, the use of gemcitabine plus S-1 (GS) therapy as a neoadjuvant chemotherapy (NAC) significantly prolonged overall survival(6, 7). GS therapy is recommended as NAC for treating resectable pancreatic cancer according to the 2022 Clinical Practice Guidelines for Pancreatic Cancer in Japan(8).

In the 1980s and 1990s, radical cure was prioritized for surgical resection, and extended lymph node and plexus dissection was performed for resectable pancreatic cancer. However, several randomized controlled trials (RCTs)(9, 10) showed that extended lymph node dissection in pancreatic cancer patients did not improve prognosis and these findings led to the recommendation against performing extended lymph node dissection(8). While extended lymph node dissection has been ruled out for treatment, the optimal range of standard lymph node dissection in the head, body or tail of the pancreas has not been reported on. Moreover, the latest guidelines from the National Comprehensive Cancer Network and Japan Pancreas Society do not specify the appropriate station for lymph node dissection.

The index of estimated benefit of lymph node dissection(11) is calculated as the metastasis rate × survival rate of metastatic patients, which is useful in deciding whether to perform lymph node dissection. This index is used to predict the effect of each lymph node dissection in patients with gastric cancer(11); however, it cannot be accurately calculated when there is variation in the area and precision of dissection, so retrospective studies are unable to accurately assess the benefit of dissection. The aim of this prospective trial is to determine the optimal station to perform lymph node dissection during pancreatectomy for PDAC patients after NAC. This will be achieved by evaluating the rate of lymph node metastasis and the index of estimated benefit of each type of lymph node dissection.

# 4．Objective

# The aim of the LYMRIN trial is to determine the optimal range of lymph node dissection for patients with resectable pancreatic cancer after NAC. This will be achieved by clarifying the index of estimated benefit of lymph node dissection, which will be calculated by the metastasis rate of each lymph node station and the survival rate of the patient with lymph node metastasis.

# 5．Basis of scientific rationality and significance

Pancreatic cancer deaths are increasing, and the disease has an extremely poor prognosis. Although multidisciplinary treatment is performed with the aim of surgical resection to achieve R0 resection, the extent of lymph node dissection needs to be determined without excess or deficiency in order to further improve prognosis.

The aim of this study was to determine the exact percentage of metastases in regional lymph nodes and the effect of dissection in surgical treatment after preoperative adjuvant chemotherapy for resectable pancreatic cancer, and to determine the optimal extent of lymph node dissection. It is hoped that this study could provide an important medical basis for establishing a standard operating procedure.

# 6．Inclusion criteria/ Exclusion criteria

## 6.1．Inclusion criteria

1. Diagnosis：one of the following ① to ③ is fulfilled.
2. Histological diagnosis as PDAC (adenocarcinoma, adenosquamous carcinoma)

②　Cytological diagnosis as PDAC (Class IV or Class V)

③　Clinically diagnosed as PDAC from contrast-enhanced CT or MRI, although no pathological diagnosis has been made.

2) The primary tumor is diagnosed as resectable

3) No history of upper abdominal surgery

4) Surgical resection with regional lymph nodes (Table 2) dissection is possible with imaging

5) Peritoneal washing cytology: negative (if applicable)

6) Neoadjuvant chemotherapy was performed

7) Tumors determined to be suitable for radical primary resection within 28 days after neoadjuvant chemotherapy

8) Planned pancreatoduodenectomy for pancreatic head cancer, distal pancreatectomy for pancreatic body-tail cancer

9) Age: 18 years or older

10) Performance status (Eastern Cooperative Oncology Group scale): 0-1

11) Ability to understand and willingness to sign written informed consent document

6.2．Exclusion criteria

1) Active multiple primary cancers (synchronous or asynchronous within 5 years). However, a history of cancer equivalent to a 5-year relative survival rate of 95% or more, such as stage I prostate cancer, stage 0/I laryngeal cancer with a complete response to radiotherapy, or cancer of the following pathological stages that has been completely resected, even if disease-free for less than 5 years, is not included in active multiple/polycystic cancers.

Gastric cancer : Stage 0 -I, Colon cancer : Stage 0-I, Rectal cancer : Stage 0 -I, Esophageal cancer : Stage 0、Breast cancer (non-invasive) : Stage 0, Breast cancer (invasive, Paget) : Stage 0 -IIA, Uterine body cancer : Stage I, Prostate cancer : Stage I-II, Cervical cancer: Stage 0, Thyroid cancer: Stage I-III, Renal cancer: Stage I

2) Serious comorbidities (heart failure, interstitial pneumonia, renal failure, liver failure, intestinal paralysis, intestinal obstruction, poorly controlled diabetes, poorly controlled hypertension, etc.)

3) Protocol treatment (radical primary tumor resection and regional lymph node dissection) cannot be safely performed

4) Receiving pre-treatment (radiotherapy, immunotherapy, etc.) other than neoadjuvant chemotherapy

5) Pregnant or lactating women and women of childbearing potential

6) Severe psychological or neurological disease

# 7．Methods and period

7.1．Design

A multicenter prospective interventional trial


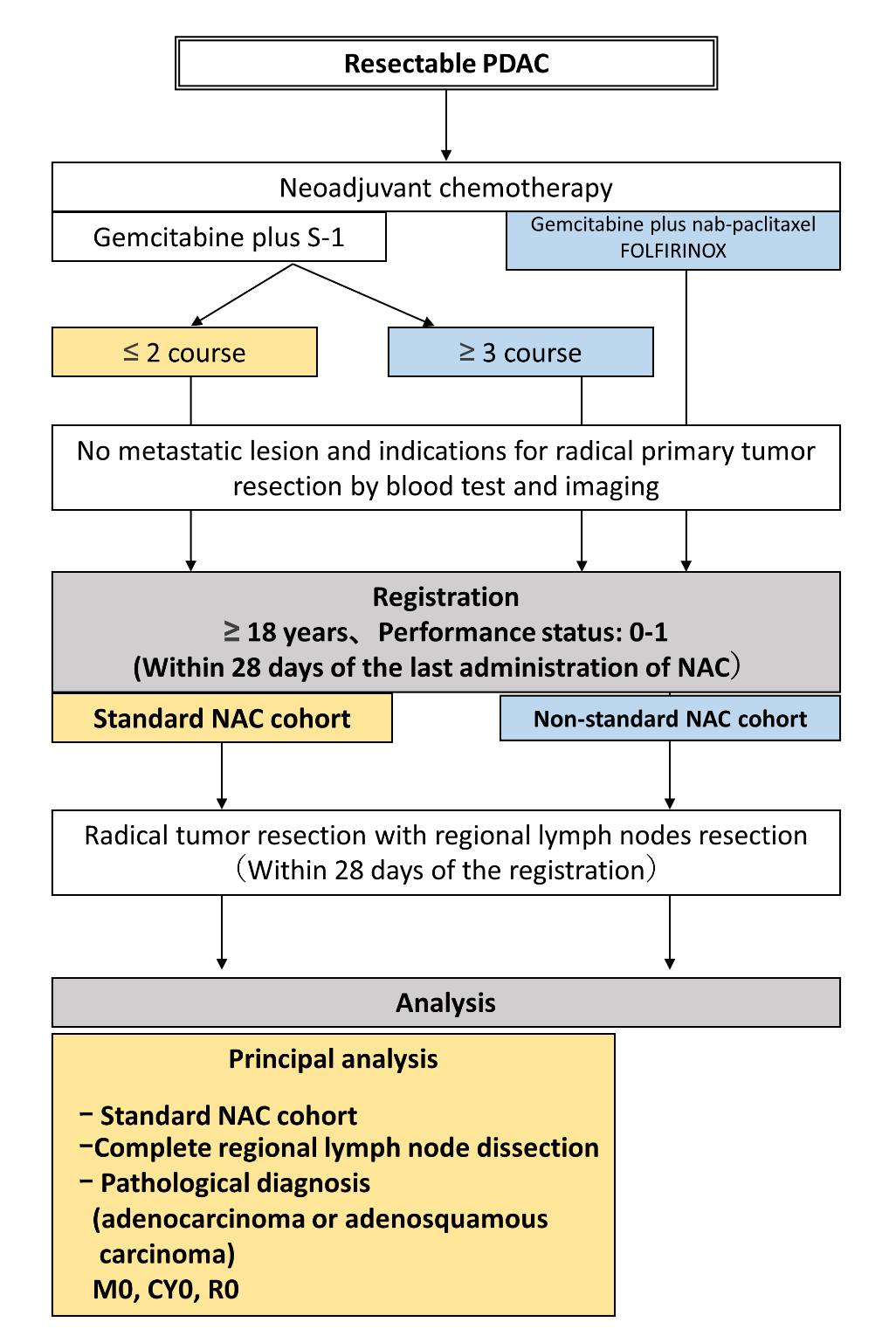
Figure 1. Flow diagram of the LYMRIN trial

For postoperative adjuvant therapy, 6 months of S-1 therapy is recommended.

However, the timing of initiation, treatment and types of postoperative adjuvant therapy are not limited.

7.2．Methods

7.2.1.　Subjects

Patients receiving preoperative chemotherapy for resectable pancreatic cancer who meet the eligibility criteria and do not meet the exclusion criteria.

7.2.2　Institutional review board (IRB)

The principal investigator consults the Ethics Review Committee, which collectively reviews the appropriateness of conducting the research. The review request process will be coordinated with the principal investigators at each institution. The principal investigator submits the documents submitted to the Institutional Ethical Review Committee and the documents required by the president of the research institution, and obtains permission to conduct the relevant research.

7.2.3.　Registration

The principal investigator will explain the study to patients who meet the eligibility criteria and do not meet the exclusion criteria, and obtain written informed consent from the patients themselves.

1. Registration is to be performed via the Electrical Data Capture (EDC) system (REDCap®). The date of registration is defined as the date the registration center is notified.
2. Patients who are scheduled to receive the standard neoadjuvant chemotherapy “gemcitabine + S-1 combination therapy” (“GS therapy”) will be enrolled in the standard NAC cohort if they have completed 2 courses or less of NAC GS therapy, undergo diagnostic imaging and blood tests to evaluate efficacy, and are judged to be eligible for radical primary tumor resection without new lesions or distant metastases. Registration is required within 28 days of the last dose of NAC.
3. Patients who received other preoperative chemotherapy (gemcitabine plus nab-paclitaxel, FOLFIRINOX (original or variant), or 3 or more courses of GS therapy) will be enrolled as a nonstandard cohort after similar evaluation.
4. After the registration screen verifies that the eligibility requirements have been met, a registration number will be issued. At this point, "registration" is considered complete. If the input data is insufficient or does not meet the eligibility criteria, a registration number will not be issued, and "Registration" will not be completed.
5. Postoperative adjuvant chemotherapy for enrolled patients is recommended to be 6 months of S-1 therapy as the standard therapy, but whether adjuvant therapy is indicated, the treatment regimen, and the dosage allows each patient to decide.
6. The number of patients in the standard NAC cohort who meet the primary analysis criteria will be the planned enrollment number. Once the planned number of enrollments is reached, new enrollments will be closed. Patients who are enrolled at this time but have not yet received a radical primary resection will also be included in the analysis.

7.2.4.　Perioperative chemotherapy

7.2.4.1.　Neoadjuvant chemotherapy (NAC)

7.2.4.1.1.　Standard NAC cohort

According to the results of the Prep-02 / JSAP-05 trial, two courses of GS therapy are the standard neoadjuvant chemotherapy for resectable pancreatic cancer in Japan(6). Patients who have completed no more than two courses (no more than four doses of gemcitabine hydrochloride) of preoperative GS therapy (with or without completion) as neoadjuvant treatment will be analyzed as the standard NAC cohort.

7.2.4.1.2.　Non-standard NAC cohort

Patients treated with gemcitabine+nab-paclitaxel, FOLFIRINOX (original or modified) or three or more courses of GS therapy will be analyzed as a non-standard cohort.

7.2.4.2　Adjuvant chemotherapy

The presence or absence of adjuvant therapy, regimen and duration of administration are not specified.

7.2.5.　Treatment protocol

7.2.5.1.　Treatment protocol

Treatment protocol in this study is defined as radical primary pancreatectomy (pancreaticoduodenectomy or distal pancreatectomy) and regional lymph node dissection from the start to the end of surgery.

7.2.5.2. Primary tumor resection

Primary tumor resection is performed within 28 days of registration. Surgical resection in this study allows open, laparoscopic and robot-assisted pancreatectomy.

Immediately after laparotomy or laparoscopic insertion, search for distant metastases, such as liver or peritoneal dissemination. If distant metastases are suspected intraoperatively, sampling and assessment by intraoperative rapid pathology is recommended. Peritoneal cytology is required at the time of resection surgery, but rapid diagnosis is not mandatory.

If no distant metastases are present and the tumor is considered resectable, the primary tumor should be resected according to the following surgical technique depending on the location of the tumor with regional lymph node dissection as indicated in ‘7.2.5.3.’ Table 1. Number 16 lymph node dissection for staging is not required.

・Pancreatic head cancer: pancreatoduodenectomy

・Pancreatic body cancer: distal pancreatectomy

・Pancreatic tail cancer: distal pancreatectomy

Pancreatoduodenectomy for pancreatic body cancer is not acceptable and included in the analysis as a deviation of treatment protocol. Patients who underwent total pancreatectomy or major artery resection (celiac artery, hepatic artery, variant hepatic artery, superior mesenteric artery) are similarly not included in the analysis. The border between the pancreatic head and body is the left lateral margin of the superior mesenteric vein and portal vein. Uncinate process is included in the head. The border between the pancreatic body and the tail is the left lateral margin of the aorta. If the tumor spreads over two sites, the main area is the site of tumor occupation.


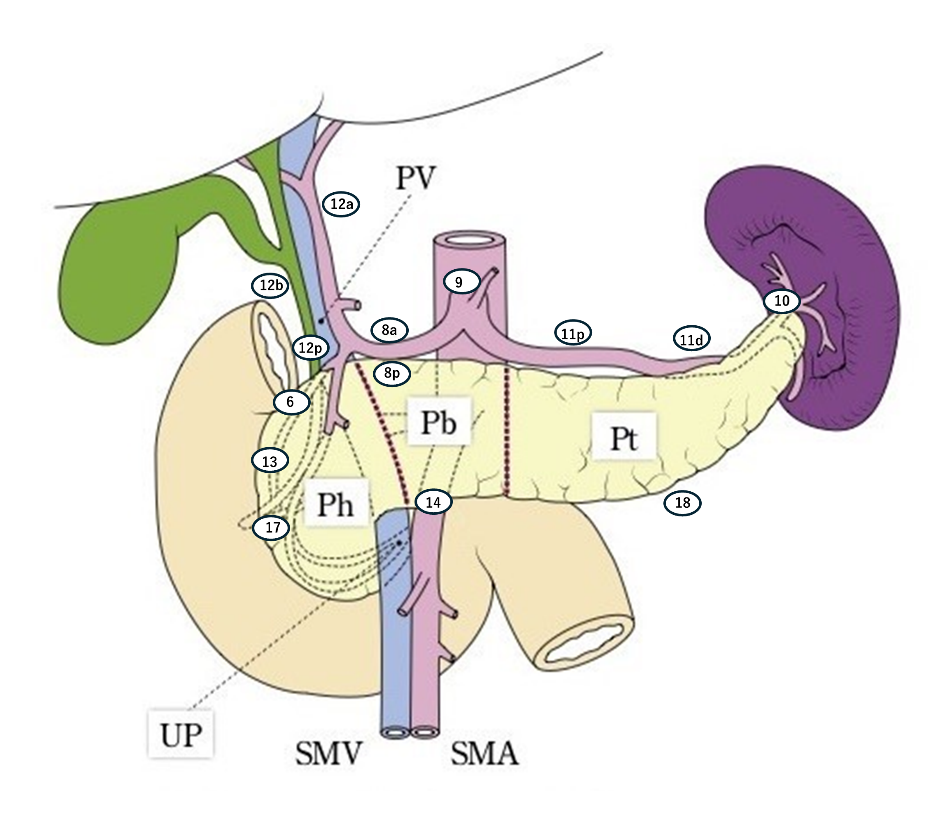


Figure 2. Location of the pancreas（From General rules for the study of pancreatic cancer, the 8th edition(1)）

7.2.5.3.　Regional lymph nodes resection

In accordance with General rules for the study of pancreatic cancer 8^th^ edition(1), the following regional lymph nodes dissection should be performed according to the site of the tumor location (Figure 2-4). Dissection of non-regional lymph nodes is acceptable for safe intraoperative manipulation and vascular taping. Dissection of lymph node 16 for the purpose of securing the dorsal pancreatic margin is acceptable. Even if it is not possible to perform all the lymph node dissection shown in Table 1, the patient will be included in the analysis and will be followed up.

**Table 1. Regional lymph nodes by tumor location**

| Tumor location of pancreas | Regional lymph nodes |
| --- | --- |
| Head | 6, 8a, 8p, 12a, 12b, 12p, 13, 14t,14op, 17 |
| Body | 8a, 8p, 9, 10, 11p, 11d, 14t, 18 |
| Tail | 8a, 9, 10, 11p, 11d, 18 |

Station numbers and names of lymph nodes related to the pancreas.

| **Number** | **Name** |
| --- | --- |
| 1 | Right cardial lymph nodes |
| 2 | Left cardial lymph nodes |
| 3 | Lymph nodes along the lesser curvature of the stomach |
| 4 | Lymph nodes along the greater curvature of the stomach |
| 5 | Suprapyloric lymph nodes |
| 6 | Infrapyloric lymph nodes |
| 7 | Lymph nodes along left gastric artery |
| 8a | Lymph nodes in the anterosuperior group along common hepatic artery |
| 8p | Lymph nodes in the posterior group along common hepatic artery |
| 9 | Lymph nodes around celiac artery |
| 10 | Lymph nodes at the splenic hilum |
| 11p | Lymph nodes along the proximal splenic artery |
| 11d | Lymph nodes along the distal splenic artery |
| 12a | Lymph nodes along the hepatic artery |
| 12p | Lymph nodes along portal vein |
| 12b | Lymph nodes along the bile duct |
| 13 | Lymph nodes on the posterior aspect of the head of the pancreas |
| 14t | Lymph nodes along the superior mesenteric artery; tumor side |
| 14op | Lymph nodes along the superior mesenteric artery; opposite side of tumor |
| 15 | Lymph nodes along middle colic artery |
| 16a | Lymph nodes around the abdominal aorta a |
| 16b | Lymph nodes around the abdominal aorta b |
| 17 | Lymph nodes on the anterior surface of the head of the pancreas |
| 18 | Lymph nodes along the inferior margin of the pancreas |


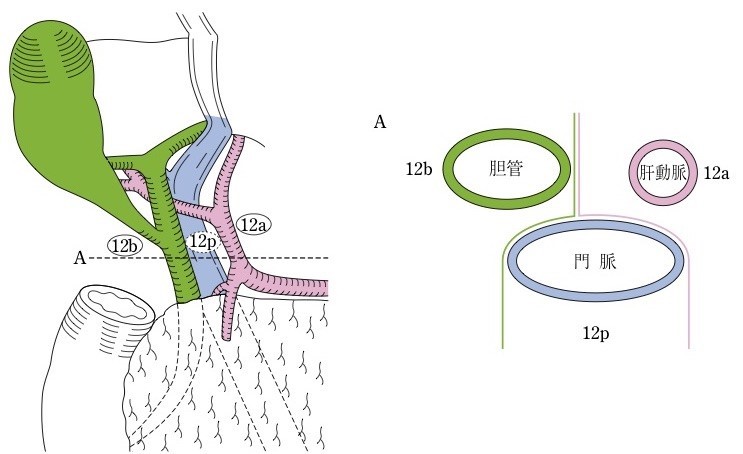


HA

CBD

PV

PV

Figure 3．Hepatoduodenal ligament and lymph nodes


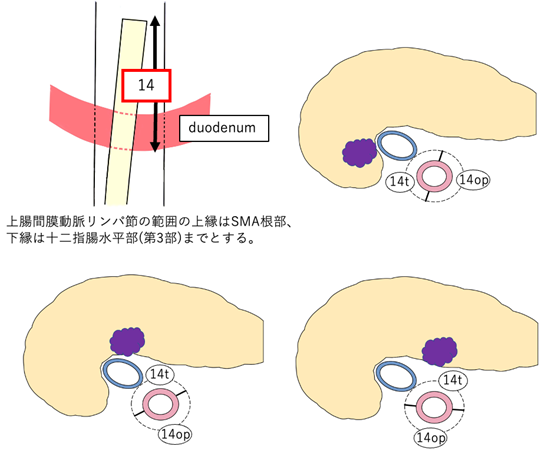


Figure 4．Superior mesenteric artery and lymph nodes

7.2.5.4.　The methods of gastrointestinal tract reconstruction

No stipulation

## 7.3．Endpoints

### 7.3.1．Primary endpoint

This clinical trial will primarily evaluate the metastatic rate of each lymph node station in a standard cohort treated with NAC.

### 7.3.2．Secondary endpoints

　 The secondary endpoints include the index of estimated benefit of each lymph node dissection (metastatic rate x 3- or 5-year survival), disease-free survival, overall survival, cancer-specific survival, postoperative complications, the location of the relapse site in the standard and nonstandard cohorts, and the metastatic rate of each lymph node station in the nonstandard cohort. The results will be published in a conference or in a paper.

### 7.3.3．Definitions of evaluation points

### ①Percentage of metastasis in each lymph node

### In each lymph node: The patients with positive lymph node / All patients in the analysis

The patients with no dissected lymph node are not included in denominator of metastatic rate.

1. The index of estimated benefit of each lymph node dissection

(Metastatic rate) × (3-year disease free survival) or (Metastatic rate) × (5-year overall survival) (11).

③ Disease free survival

The period beginning with the date of surgery and ending with the earlier of recurrence or the date of death from any cause. Last day of clinically confirmed absence of recurrence in survivors without recurrence.

Metachronous primary cancers will not be terminated, and the patient will be considered disease free survival until pancreatic cancer recurrence is observed.

④ Disease free survival rate

Percentage of patients alive without recurrence or death from any cause at 1, 2, 3, 4, and 5 years from the date of surgery

⑤ Overall survival

The period beginning on the date of the start of neoadjuvant chemotherapy and ending on the date of death from any cause. Survival cases are terminated on the date of last confirmation of survival. Untraceable cases are terminated on the last date of confirmed survival before loss to follow-up.

⑥ Overall survival rate

Percentage of patients alive without death from any cause at 1, 2, 3, 4, and 5 years from the start of neoadjuvant chemotherapy.

⑦ Cancer specific survival

The period beginning on the date of the start of preoperative chemotherapy and ending on the date of death due to pancreatic cancer.

⑧ Cancer specific survival rate

Percentage of survivors who have not died from pancreatic cancer at 1, 2, 3, 4, and 5 years from the start of neoadjuvant chemotherapy

⑨ Postoperative complication

All surgical cases will be used as the denominator, and intraoperative, early postoperative (until initial discharge), and late postoperative (after initial discharge) adverse events will be graded using the Clavien-Dindo classification(12) (Grade II or higher) or the ISGPS (International Study Group of Pancreatic Surgery) classification(13-15), respectively.

⑩ Pattern of recurrence

Recurrence pattern and frequency.

## 7.4．Statistical analysis

- - 1. Principal analysis requirements

1. Radical resection within 28 days of registration
2. Pancreatectomy with regional lymph nodes dissection shown in Table 1.
3. Pathological examination diagnoses invasive ductal adenocarcinoma (adenocarcinoma, adenosquamous carcinoma). R0 resection is required. R0 is defined as the absence of cancer cells within 1mm of the edge by Royal College of Pathology(2).
4. Abdominal cytology negative.
5. No distant metastasis.
6. Not all of the following
7. Major artery resection and reconstruction
8. Surgical mortality

### 7.4.2．Full Analysis Set (FAS)

Patients who meet the criteria specified in 7.4.1. in the standard NAC cohort patients will be the population to be analyzed.

7.4.3.　Other analysis

In the non-standard cohort patients and in the standard NAC cohort patients who do not meet the criteria for the main analysis, statistical analysis will be performed as in the main analysis.

### 7.4.4．Procedures for handling missing, rejected and anomalous data

In case of missing data, the data center will contact each institution to ensure that no data are missing. After the study is completed, a final decision on data handling will be made at a case review meeting between the principal investigator, statistical analysts, and data managers, and analysis will be conducted after the data have been fixed.

### 7.4.5．Analysis of evaluation points

### ① Percentage of metastasis in each lymph node

Calculate the percentage of metastases in each lymph node for FAS and estimate Wilson's 95% confidence interval for the mother proportion. Non-dissected cases are not included in the denominator of the metastatic proportion.

② The index of estimated benefit of each lymph node dissection

Calculate the index of estimated benefit of each lymph node dissection for FAS. Non-dissected cases are not included in the denominator of the metastasis rate.

③ Disease free survival

④ Disease free survival rate

⑤ Overall survival

⑥ Overall survival rate

The Kaplan-Meier method is used to estimate the survival curves from ③ to ⑥. The estimated survival curves are plotted graphically. The 95% confidence interval of the annual survival rate against the survival curve is estimated using the Greenwood formula, and the confidence interval of the median survival time is estimated using the Brookmeyer and Crowley method.

⑦ Cancer specific survival

⑧ Cancer specific survival rate

For cancer specific survival analysis in ⑦ and ⑧, a competing risk analysis will be performed with death from non-pancreatic cancer as the competing event and death from pancreatic cancer as the event of interest. Cumulative incidence will be estimated and comparisons of group differences will be made using the Gray test.

⑨ Postoperative complication

Calculate the grade and incidence of intraoperative and early postoperative complications (up to the first discharge from the hospital) for all surgical cases. A 95% confidence interval will be estimated for the incidence rate. Logistic regression analysis (multivariate analysis) will be used to explore risk factors. Odds ratios and 95% confidence intervals based on the regression parameters for each factor are calculated. Results from variable selection using the regression stepwise method will be annexed.

## 7.5．Sample size estimation and the rationale for setting the number of registrations

### 7.5.1．Sample size estimation

The expected enrollment for this trial is 545 patients in the standard NAC cohort (200 patients with pancreatic head cancer, 165 patients with pancreatic body cancer, and 180 patients with pancreatic tail cancer). Registration of sites that have reached the planned number of registrations will be considered complete, and only those sites that have not reached the planned number of registrations will continue to be registered.

### 7.5.2．The rationale for setting the number of registrations

The number of patients, the lymph nodes (pancreatic head: no. 14, pancreatic body: no. 10, and pancreatic tail: no. 8a) will be selected because they are the most controversial in terms of dissection or not selected due to the low frequency of lymph node metastasis. The metastasis rate of each patient will be used as the basis for calculation. The Pancreatic Cancer Registry Database in the National Clinical Database have reported that the percentage of pathologically positive lymph node No. 14 for pancreatic head cancer is 10.9%. The Wilson score method was used to calculate the number of patients to be enrolled to achieve a probability of 90% or greater that the metastatic rate would fall within the 95% confidence interval of ±5%. The number of enrolled patients was estimated to be 183. Considering that approximately 10% of patients may be excluded from the analysis due to ineligibility, the target enrollment number in the No. 14 lymph node is set at 200 patients. Similarly, for patients with pancreatic body cancer, we estimated 151 patients will be required with a metastatic rate of 3.5% in the No. 10 lymph node and a 95% confidence interval width of at least 80% for the probability of being within 3.5%. Considering that approximately 10% of patients will be excluded from the analysis, the target enrollment number is set at 165 patients. For patients with pancreatic tail cancer, the percentage of pathologically positive No. 8a lymph nodes is 3.2%. The number of cases required to achieve a probability of 80% or greater within the 95% confidence interval of ±3.2% is estimated to be 166. Therefore, the target number of enrollment is set at 180 patients.

### 7.5.3．Sample size estimation in non-standard NAC cohort

Enrollment of the non-standard cohort will be terminated when the number of patients meeting the primary analysis criteria in all sites (head, body, tail) is completed in standard NAC cohort.

## 7.6．Study period

Registration: 3 years（the date of approval - September 30, 2026）

Follow-up period: 5 years after the end of the registration period

Total study period: 8 years（the date of approval - September 30, 2031）

# 8．Evaluation ・Clinical examination・Schedule

8.1.　Before registration

8.1.1.　Before registration

1）Age

2）Sex

3）Comorbidities, pre-existing conditions, surgical history

4）Abdominal cytology (if screening laparoscopy is performed)

5）HBs antigen, HBc antibody, HBs antibody, HCV antibody

8.1.2.　 Examination to be performed within 28 days prior to registration

1）Contrast-enhanced chest, abdominal and pelvic CT

・Images scanned at other hospitals are not acceptable.

・Method: Using an intravenous contrast agent, imaging with a multidetector-row computed tomography (MDCT) imaging system

・slice：under 5mm

・For the chest, non-contrast CT is acceptable.

2）Resting 12-lead ECG

8.1.3.　 Examination to be performed within 14 days prior to registration

1）General condition: Performance Status（ECOG）、height、body weight

2）CBC: leukocyte, neutrophil, lymphocyte, hemoglobin, platelet

3）Biochemistry tests: total protein, albumin, total bilirubin, AST, ALT, creatinine, sodium, potassium, C-reactive protein

4）Tumor markers: CEA、CA19-9、DUPAN-2

8.2.　After registration

8.2.1.　Surgical findings

1) date of surgery, surgical time

2) surgical methods, portal vein resection and reconstruction, combined organ resection, pancreatectomy completed, lymph nodes dissection, the reason of unresectable tumor (liver metastasis, peritoneal dissemination, positive for extra regional lymph nodes, positive for abdominal cytology, and so on).

3) Estimated blood loss, transfusion

4) Intraoperative complication

8.2.2. Pathological findings

1) Histological type

2) Tumor location, count, diameter

3) Local progression (T), lymph nodes metastasis (N), distant metastasis (M), peritoneal cytology (CY)

4) Local progression（portal vein, artery, extrapancreatic plexus invasion, stomach, colon, adrenal gland）

5) Curability（R0、R1、R2）

6) Lymph nodes metastasis in each

8.2.3．Postoperative evaluation

1) Day of discharge

2) Re-operation

3) CBC：leukocyte, neutrophil, lymphocyte, hemoglobin, platelet

4) Biochemistry tests：total protein, albumin, total bilirubin, AST, ALT, creatinine, sodium, potassium, C-reactive protein

5) Early postoperative complication：From the end of surgery to 30 days postoperatively. The physician's judgment regarding the causal relationship to the surgery will also be reported.

8.2.4.　Postoperative evaluation after 31days

After the 31st postoperative day, the patient should be evaluated at the following frequency.

31 days to 3 years: Every 3 months. Allow up to 1 month before and after.

3 to 5 years: Every 6 months. Allow up to 2 months before and after.

5 years and up: Every 1 year. Allow up to 3 months before and after.

8.2.4.1.　Safety assessment

1) Postoperative late complications (after 31 days postoperatively)

The physician's judgment regarding the causal relationship to treatment is also entered into the CRF.

2) Information of adjuvant therapy

8.2.4.2.　Efficacy assessment

1) Contrast-enhanced chest, abdominal and pelvic CT

Contrast-enhanced MRI is acceptable if the patient did not have an allergy to CT contrast at the time of registration but developed an allergy to CT contrast after registration. Simple CT and MRI are also acceptable if renal dysfunction, bronchial asthma, or other factors preclude the use of contrast-enhanced examination.

2) Tumor markers：CEA、CA19-9、DUPAN-2

8.2.4.3.　Follow-up

Follow-up will be conducted up to 5 years after the end of enrollment in the study, so individual patients will continue to report after 5 years of enrollment according to the CRF cutoff date.

1) Confirmation of outcome

Evaluate for death from any cause. In cases of death, record whether the death was due to pancreatic cancer or another cause.

2) Confirmation of recurrence

If recurrence is observed, record the pattern of recurrence.

8.3.　Study schedule

|  | Before registration | | | Surgery | After registration | | | | |
| --- | --- | --- | --- | --- | --- | --- | --- | --- | --- |
|  | Within  28 D | Within  14 D | Before  Surgery |  | Pathological  Diagnosis | Within  30 D | 31 D  -  3 Y | 3 Y  - 5 Y | 5 Y |
| Informed consent |  |  | ● |  |  |  |  |  |  |
| Registration |  |  | ● |  |  |  |  |  |  |
| Backgrounds |  |  | ● |  |  |  |  |  |  |
| Blood tests |  | ● |  |  |  | ● | ○ | ○ | ○ |
| Tumor markers |  | ● |  |  |  |  | ◎^3M^ | ◎^6M^ | ◎^12M^ |
| General condition |  | ● |  |  |  |  | ○ | ○ | ○ |
| Contrast-enhanced CT | ● |  |  |  |  |  | ◎^3M^ | ◎^6M^ | ◎^12M^ |
| ECG | ● |  |  |  |  |  |  |  |  |
| Peritoneal cytology |  |  | ○ | ● |  |  |  |  |  |
| Surgical information |  |  |  | ● |  |  |  |  |  |
| Complication |  |  |  | ● |  | ● | ◎^3M^ | ◎^6M^ | ◎^12M^ |
| Pathological findings |  |  |  |  | ● |  |  |  |  |
| Adjuvant therapy |  |  |  |  |  |  | ◎^3M^ | ◎^6M^ | ◎^12M^ |
| Survival information |  |  |  |  |  |  | ◎^3M^ | ◎^6M^ | ◎^12M^ |

●：Required　○：Recommended

◎^3M、6M, 12M^：3, 6, 12 months after surgery

# 9．Informed consent

### 9.1.1．Explanation to patients

1. Title of study and permission of responsible research institution

2. Research institutions and principal investigator

3. Aim

4. Methods and period

5. Study participants

6. Risks and benefits expected to result from participation in the study

7. Consent and withdrawal

8. Methods of disclosure of study information

9. Disclosure of study protocols and other materials

10. Handling of personal information

11. Methods of information preservation and disposal

12. Status of conflicts of interest

13. Financial burden or gratuity for treatment and examination

14. Explanation of the treatment the patient would receive if not enrolled in the study

15. Explanation that post-treatment after completion of treatment protocol treatment is also appropriate

16. Compensation for adverse health effects caused by the research

17. Possible use of research data for future research (secondary use)

18. Access to research data, medical records, etc.

19. Declaration of consent for participation

20. How to inquire about this study

# 10．Methods of handling, storing and disposing of personal information.

All researchers involved in this research should conduct this research in accordance with the “Declaration of Helsinki” and the “Ethical Guidelines for Life Sciences and Medical Research Involving Human Subjects”. We recognize that information related to privacy, including personal information, should be strictly protected and handled with care based on the principle of respect for the individual personality. The reseracheres will take all possible management measures to protect privacy. Personal information obtained from this research should not be leaked without justifiable reason.

Rgistration numbers are used to identify and refer to registered subjects.

To identify anonymized registered subjects when necessary, a consolidated table of registration numbers and registered patients will be prepared at the time consent is obtained.

Electronic data stored in the Electronic Data Capture (EDC) system will be stored on a secure cloud server. Only those who are authorized by the principal investigator will be given access rights, and a list of access rights will be created.

Information related to this study will be retained until the later of five years after the completion of the entire study or three years after the final publication of the results. Thereafter, the information will be disposed of in an unrecoverable form by cutting paper media or erasing electronic files.

# 11．Comprehensive assessment of the burden, risks and benefit to the research patients.

Although protocol treatment does not deviate from the usual surgical procedures, the possibility of additional invasiveness cannot be ruled out due to the goal of ensuring lymph node removal. Adverse events are monitored by the monitoring staff to determine whether they are within the expected range, and any serious or unanticipated adverse events are carefully reviewed. The Efficacy and Safety Evaluation Committee may consider discontinuation of enrollment or the entire study. In principle, health insurance and the patients will pay the costs of treatment for any adverse health effects caused by this clinical study. In addition, the number of hospital visits, examinations, and the amount of blood sampling may increase compared to the usual medical treatment.

11.1.　Expected adverse events

Adverse events are defined as any unfavorable signs (including abnormal laboratory test results), symptoms, or illnesses that occur in the patients from the start of surgery to 30 days after the end of surgery, regardless of causal relationship to the protocol treatment. Postoperative complications will be graded using the Clavien-Dindo classification(12) or the ISGPS (International Study Group of Pancreatic Surgery) classification(13-15).

11.1.1.　Intraoperative expected adverse events

Hemorrhage, thrombosis/embolism, acute coronary syndrome/myocardial infarction, supraventricular tachycardia, atrial fibrillation, ventricular arrhythmia, atrial flutter, cerebrovascular ischemia, fever, hypothermia, gastrointestinal bleeding, intraoperative hepatobiliary system/ spleen/ endocrine system/ artery/ vein/ lymph/ digestive tract/ respiratory system/ nervous system injury, allergic reaction

11.1.2.　Post operative expected adverse events

Postoperative bleeding, pancreatic fistula, intra-abdominal abscess, aneurysm, ruptured aneurysm, delayed gastric emptying, fever, chills, hypothermia, anorexia, dehydration, nausea, vomiting, sweating, hypothermia, weight loss, lateral abdominal pain, back pain, myalgia, neck pain, gastric bleeding, gastric stricture, gastric perforation, gastric necrosis, gastric obstruction, gastrointestinal bleeding, gastrointestinal stenosis, gastric perforation, gastrointestinal obstruction, pancreatic hemorrhage, pancreatitis, pancreatic necrosis, peritoneal necrosis, constipation, diarrhea, intestinal obstruction, ascites, dysphagia, liver hemorrhage, cholecystitis, cholangitis, obstructive jaundice, catheter-related infection, medical device-related infection, wound infection, infective small bowel colitis, renal infection, urinary tract infection, lung infection, upper respiratory tract infection, mediastinal infection, sepsis, wound opening, incisional hernia, leak of gastrointestinal anastomosis, anastomotic ulcer, tracheal hemorrhage, pleural effusion, intrathoracic hemorrhage, pneumothorax, aspiration, atelectasis, bronchopulmonary hemorrhage, cough, hoarseness, hypoxia, stuttering, acute coronary syndrome, myocardial infarction, atrial fibrillation, flutter, ventricular arrhythmia, thromboembolism, hypertension, hypotension, hematoma, acute renal failure, hematuria, renal bleeding, urinary retention, upper limb edema and lower limb lymph edema, cerebral vascular ischemia, peripheral motor neuropathy, peripheral sensory neuropathy, confusion, depression, insomnia, increased serum bilirubin, increased alanine aminotransferase, increased aspartate aminotransferase, increased alkaline phosphatase, increased creatinine, hypernatremia, hyponatremia, hyperkalemia, sodiumemia, hyperkalemia, hypokalemia, hyperglycemia, hypoalbuminemia, disseminated intravascular coagulation, anemia

11.2.　Benefits

The treatment protocol does not deviate from the usual surgical procedure. Therefore, participation in this study will not directly benefit the research subjects. The results of the study may contribute to the advancement of medical science in the future.

# 12．Fundings and conflicts of interest

This study will be funded by the Department of Surgery and Science, Faculty of Medicine, Academic Assembly, University of Toyama. The Japan Pancreas Society and Japan Oncology Network in Hepatobiliary and Pancreatic Medicine will assume some of the costs. The status of conflicts of interest of the principal investigator will be examined by the Conflicts of Interest Management Committee of the University of Toyama prior to ethical review by the IRB.

# 13．Response to adverse events

13.1. Adverse events

13.1.1.　Serious adverse events

A serious adverse event is defined as any of the following.

1) Death

2) Disease that could lead to death

3) Diseases requiring hospitalization or extended stay in a medical institution for treatment

13.1.2.　Non-serious adverse events

Non-serious adverse events are defined as adverse events other than those judged to be “serious” and are determined by the principal investigator.

13.1.3.　 Causal relationship of adverse events to protocol treatment

The causal relationship of adverse events to the protocol treatment will be determined for the protocol treatment and the procedures involved in the study. The determination is classified into 5 categories: definite, probable, possible, unlikely, and not related. If the answer is “definite, probable, or possible”, it is defined as “causally related”. If it is judged to be either “unlikely” or “not related,” it is defined as “no causal relationship.

14．Monitoring

Central monitoring will be performed by an independent data monitoring committee. The purpose of monitoring is to ensure that the human rights and welfare of human subjects are protected in the context of clinical research, that research data are accurate, complete, and verifiable against source documents, and that clinical research is conducted in accordance with the protocol and applicable regulatory requirements. The monitoring committee reports serious adverse events to the efficacy and safety assessment committee.

References

1. Japan Pancreas Society. General rules for the study of pancreatic cancer, the 8th edition.2023.

2. Dataset for the histopathological reporting of carcinomas of the pancreas, ampulla of Vater and common bile duct, London :Royal College of Pathologists, 2010.

3. Sung H, Ferlay J, Siegel RL, Laversanne M, Soerjomataram I, Jemal A, et al. Global Cancer Statistics 2020: GLOBOCAN Estimates of Incidence and Mortality Worldwide for 36 Cancers in 185 Countries. CA Cancer J Clin. 2021;71(3):209-49.

4. American Cancer Society. Survival Rates for Pancreatic Cancer2024. Available from: https://www.cancer.org/cancer/types/pancreatic-cancer/detection-diagnosis-staging/survival-rates.html

5. National Comprehensive Cancer Network. Clinical Practice Guidelines in Oncology. Pancreatic Adenocarcinoma. Version 2.2021. <https://www.nccn.org/professionals/physician_gls/pdf/pancreatic.pdf> [

6. Unno M, Motoi F, Matsuyama Y, Satoi S, Matsumoto I, Aosasa S, et al. Randomized phase II/III trial of neoadjuvant chemotherapy with gemcitabine and S-1 versus upfront surgery for resectable pancreatic cancer (Prep-02/JSAP-05). Journal of Clinical Oncology. 2019;37(4_suppl):189-.

7. Sugiura T, Toyama H, Fukutomi A, Asakura H, Takeda Y, Yamamoto K, et al. Randomized phase II trial of chemoradiotherapy with S-1 versus combination chemotherapy with gemcitabine and S-1 as neoadjuvant treatment for resectable pancreatic cancer (JASPAC 04). Journal of Hepato-biliary-pancreatic Sciences. 2023;30(11):1249-60.

8. Okusaka T, Nakamura M, Yoshida M, Kitano M, Ito Y, Mizuno N, et al. Clinical Practice Guidelines for Pancreatic Cancer 2022 from the Japan Pancreas Society: a synopsis. International Journal of Clinical Oncology. 2023;28(4):493-511.

9. Nimura Y, Nagino M, Takao S, Takada T, Miyazaki K, Kawarada Y, et al. Standard versus extended lymphadenectomy in radical pancreatoduodenectomy for ductal adenocarcinoma of the head of the pancreas: long-term results of a Japanese multicenter randomized controlled trial. Journal of hepato-biliary-pancreatic sciences. 2012;19(3):230-41.

10. Jang JY, Kang JS, Han Y, Heo JS, Choi SH, Choi DW, et al. Long-term outcomes and recurrence patterns of standard versus extended pancreatectomy for pancreatic head cancer: a multicenter prospective randomized controlled study. Journal of hepato-biliary-pancreatic sciences. 2017;24(7):426-33.

11. Sasako M, McCulloch P, Kinoshita T, Maruyama K. New method to evaluate the therapeutic value of lymph node dissection for gastric cancer. The British journal of surgery. 1995;82(3):346-51.

12. Dindo D, Demartines N, Clavien PA. Classification of surgical complications: a new proposal with evaluation in a cohort of 6336 patients and results of a survey. Annals of surgery. 2004;240(2):205-13.

13. Bassi C, Marchegiani G, Dervenis C, Sarr M, Abu Hilal M, Adham M, et al. The 2016 update of the International Study Group (ISGPS) definition and grading of postoperative pancreatic fistula: 11 Years After. Surgery. 2017;161(3):584-91.

14. Wente MN, Bassi C, Dervenis C, Fingerhut A, Gouma DJ, Izbicki JR, et al. Delayed gastric emptying (DGE) after pancreatic surgery: a suggested definition by the International Study Group of Pancreatic Surgery (ISGPS). Surgery. 2007;142(5):761-8.

15. Wente MN, Veit JA, Bassi C, Dervenis C, Fingerhut A, Gouma DJ, et al. Postpancreatectomy hemorrhage (PPH): an International Study Group of Pancreatic Surgery (ISGPS) definition. Surgery. 2007;142(1):20-5.

**APPENDIX 1.** Institution list

| Asahikawa Medical University |
| --- |
| Chiba Cancer Center |
| Chiba University |
| Dokkyo Medical University |
| Dokkyo Medical University Saitama Medical Center |
| Fujita Health University |
| Fujita Health University Bantane Hospital |
| Hirosaki University |
| Hiroshima University |
| Hokkaido University |
| Hyogo Medical University |
| JA Onomichi General Hospital |
| Jichi Medical University |
| Jichi Medical University Saitama Medical Center |
| Juntendo University |
| Kagawa University |
| Kagoshima University |
| Kanagawa Cancer Center |
| Kansai Medical University |
| Keio University |
| Kindai University |
| Kyorin University |
| Kyoto University |
| Kyusyu University |
| Mie University |
| Miyazaki University |
| Nagoya Central Hospital |
| Nagoya University |
| Nara Medical University |
| Ohara HealthCare Foundation, Kurashiki Central Hospital |
| Osaka City General Hospital |
| Osaka International Cancer Institute |
| Osaka University |
| Saitama Cancer Center |
| Sapporo Medical University |
| Showa University |
| Tohoku University |
| Tokyo Medical University |
| University of Toyama |
| Wakayama Medical University |
| Yamagata University |
| Yamaguchi University |
