# Supplementary material for: A multicenter prospective study to determine the optimal range of lymph node dissection in pancreatic cancer surgery after neoadjuvant chemotherapy (LYMRIN-Trial): Project study by the Japan Pancreas Society and JON 2302-P
Source: PLoS One. 2025 Jun 17;20(6):e0325667. doi: 10.1371/journal.pone.0325667 (PMC12173190; doi:10.1371/journal.pone.0325667)
Supplement: Attachment — Submitted filename: Response to Reviewers R1.docx [file pone.0325667.s005.docx]

Response to the comments of the editor and the reviewers

Editor

Thank you for reviewing and handling our manuscript entitled " A multicenter prospective study to determine the optimal range of lymph node dissection in pancreatic cancer surgery after neoadjuvant chemotherapy (LYMRIN-Trial): Project study by the Japan Pancreas Society and JON 2302-P". We re-wrote the manuscript, tables, and supplementary files according to reviewers’ comments. We also responded to the Journal requirements one by one. The manuscript was brushed up. We have marked in red the changes made in the manuscript.

Journal Requirements:

1. [*Please ensure that your manuscript meets PLOS ONE's style requirements, including those for file naming. The PLOS ONE style templates can be found at*

*https://journals.plos.org/plosone/s/file?id=wjVg/PLOSOne_formatting_sample_main_body.pdf and* [*https://journals.plos.org/plosone/s/file?id=ba62/PLOSOne_formatting_sample_title_authors_affiliations.pdf*](https://journals.plos.org/plosone/s/file?id=ba62/PLOSOne_formatting_sample_title_authors_affiliations.pdf)]

We rewrote the manuscript in accordance with the PLOS ONE style and Study Protocol Article templates.

2. [*We note that the grant information you provided in the ‘Funding Information’ and ‘Financial Disclosure’ sections do not match*.]

As the editor said, we rewrote the funding information and matched ‘Funding Information’ and ‘Financial Disclosure’.

3. [*Thank you for stating the following financial disclosure:*

*"Committee for the Promotion of clinical research of the Japan Pancreas Society"*

*Please state what role the funders took in the study. If the funders had no role, please state: ""The funders had no role in study design, data collection and analysis, decision to publish, or preparation of the manuscript."" If this statement is not correct you must amend it as needed. Please include this amended Role of Funder statement in your cover letter; we will change the online submission form on your behalf.*]

We added the funder statement in the manuscript and cover letter as follows. The funder had no role in this study.

The funders had no role in study design, data collection and analysis, decision to publish, or preparation of the manuscript.

(Page3, line 37)

4. [*Your ethics statement should only appear in the Methods section of your manuscript. If your ethics statement is written in any section besides the Methods, please move it to the Methods section and delete it from any other section. Please ensure that your ethics statement is included in your manuscript, as the ethics statement entered into the online submission form will not be published alongside your manuscript.*]

We moved the ethics statement into the Methods section.

(Page 14, line 255)

5. [*Please include a copy of Table 1 and 2 which you refer to in your text on page 8 and 11.*]

We include a copy of Table 1 and 2 directly after the paragraph in which they are first cited.

6. [*Please include your tables as part of your main manuscript and remove the individual files. Please note that supplementary tables (should remain/ be uploaded) as separate ""supporting information"" files*]

We include Tables as part of your main manuscript and removed the individual files.

7. [*Please include captions for your Supporting Information files at the end of your manuscript, and update any in-text citations to match accordingly. Please see our Supporting Information guidelines for more information: http://journals.plos.org/plosone/s/supporting-information.*]

We added the captions of Supporting information at the end of the manuscript.

8. [*Please review your reference list to ensure that it is complete and correct. If you have cited papers that have been retracted, please include the rationale for doing so in the manuscript text, or remove these references and replace them with relevant current references. Any changes to the reference list should be mentioned in the rebuttal letter that accompanies your revised manuscript. If you need to cite a retracted article, indicate the article’s retracted status in the References list and also include a citation and full reference for the retraction notice.*]

We reviewed the reference list to ensure that it is complete and correct.

Reviewer 1:

Thank you for your important suggestion. We totally agree with your professional comments.

#1. [*This study seems prospective observational trial because the treatment included in the study is a standard treatment for resectable PDAC and participants will not receive any additional intervention.*]

We appreciate the professional comments. As the reviewer said, neoadjuvant GS therapy and surgical resection with dissection of regional lymph nodes are standard treatments for resectable PDAC. However, this study includes several requirements with additional interventions. In the pancreas head tumor, No. 14 op lymph node (Lymph nodes along the superior mesenteric artery; opposite side of tumor) is not a regional lymph node in General rules for the study of pancreatic cancer, 8^th^ edition, but our protocol needs to dissect No. 14op lymph nodes with pancreatoduodenectomy. Although the treatment protocol does not deviate from the standard surgical procedures, the possibility of additional invasiveness cannot be ruled out due to achieving certain lymph nodes dissection. We determined that these additional invasions could occur as an intervention and developed a protocol. We added the comments in the discussion as follows.

‘Although NAC-GS therapy and surgical resection with regional lymph nodes resection are standard treatments for resectable PDAC, the study was considered an interventional trial because the possibility of additional invasiveness cannot be excluded when performing definitive lymph node dissection or contralateral lymph node dissection of the SMA in the pancreatic head.’

(Page 18, line 340)

2. [*How are the numbers of lymph node stations determined? Are they determined by surgeons in the surgical fields and separately submitted, or by pathologists in the specimens?*]

We appreciated the comment. This protocol does not specify how to determine lymph node stations. The methods of determination are assumed to be performed by the surgeon dividing in the surgical fields, or by surgeons or pathologists dividing in the specimens. We added the comments in Methods.

This trial does not prescribe how to divide and determine lymph node stations.

(Page 13, line 241)

Reviewer 2:

Thank you for your important suggestion. We totally agree with your professional comments.

Major comment

1. [*Since this is a multicenter study, a more detailed set of criteria for ensuring surgical consistency should be provided. In particular, the inclusion of HBP board certification as part of the facility qualifications needs to be explicitly addressed. Additionally, any related discussion currently in the manuscript should be moved from the Discussion section to the Methods section for clarity and coherence.*]

We appreciated the professional comments. As the reviewer said, Clarification of the requirements for participating institutions is important to ensure the credibility of the trial. Institutions participating in the study are required to be an HPB board certified and perform at least 40 pancreatectomies per year. We added the comment in the Methods.

‘Institutions participating in the study are required to be an HPB board certified and perform at least 40 pancreatectomies per year.’

(Page 8, line 133)

Minor comments

1. [*Line 332, The collaborator’s name is incorrect; it should be Masafumi, not Masashi (Masafumi Imamura, from Sapporo Medical University).*]

We appreciate the comment. We rewrote the manuscript.

2. [*Lines 448, reference No. 10: This reference is a Japanese-language source, so please revise it to follow the appropriate citation format according to the journal's submission guidelines.*]

We appreciated the comments. We changed the reference.
