# Supplementary material for: A multicenter prospective study to determine the optimal range of lymph node dissection in pancreatic cancer surgery after neoadjuvant chemotherapy (LYMRIN-Trial): Project study by the Japan Pancreas Society and JON 2302-P
Source: PLoS One. 2025 Jun 17;20(6):e0325667. doi: 10.1371/journal.pone.0325667 (PMC12173190; doi:10.1371/journal.pone.0325667)
Supplement: Attachment — Submitted filename: Response to Reviewers R2.docx [file pone.0325667.s006.docx]

Response to the comments of the editor and the reviewers

Editor

Thank you for reviewing and handling our manuscript entitled " A multicenter prospective study to determine the optimal range of lymph node dissection in pancreatic cancer surgery after neoadjuvant chemotherapy (LYMRIN-Trial): Project study by the Japan Pancreas Society and JON 2302-P". We re-wrote the manuscript and table according to reviewers’ comments. We also responded to the Journal requirements one by one. The manuscript was brushed up. We have marked in red the changes made in the manuscript. We added names of collaborators on the title page.

Journal Requirements:

[Please review your reference list to ensure that it is complete and correct. If you have cited papers that have been retracted, please include the rationale for doing so in the manuscript text, or remove these references and replace them with relevant current references. Any changes to the reference list should be mentioned in the rebuttal letter that accompanies your revised manuscript. If you need to cite a retracted article, indicate the article’s retracted status in the References list and also include a citation and full reference for the retraction notice.]

We reviewed the reference list to ensure that it is complete and correct.

Reviewer #1

Thank you very much for your peer review.

[#1. In the response, the authors noted “However, this study includes several requirements with additional interventions. In the pancreas head tumor, No. 14 op lymph node (Lymph nodes along the superior mesenteric artery; opposite side of tumor) is not a regional lymph node in Japanese classification of pancreatic carcinoma by the Japan Pancreas Society: Eighth edition, but our protocol needs to dissect No. 14op lymph nodes with pancreatoduodenectomy.” If the protocol required additional interventions, they should be clearly stated in “Treatment protocol”. In addition, Table 2 included No. 14op in regional lymph nodes of the tumor in the pancreas head. As the authors noted No. 14op is not regional lymph nodes.]

We appreciate the professional comments. As the reviewer said, if additional interventions are required, we should describe the comment in treatment protocol. We added the comments below. In addition, we changed the title of Table 2 from “Regional lymph nodes by tumor location” to “Lymph nodes to be dissected by tumor location”.

This study includes several requirements with additional interventions. In the pancreas head tumor, No. 14 op lymph node (Lymph nodes along the superior mesenteric artery; opposite side of tumor) is not a regional lymph node in General rules for the study of pancreatic cancer, 8th edition [10], but our protocol needs to dissect No. 14op lymph nodes with pancreatoduodenectomy.

(Page 13, line 236)

Pancreatoduodenectomy for pancreatic head tumors requires dissection of 14op lymph node, which is outside the regional lymph node.

(Page 14, line 258)

[Line 238-240, the title of the reference should be changed.]

We appreciated the comments. We changed the title of the reference as follows:

Japanese classification of pancreatic carcinoma by the Japan Pancreas Society: Eighth edition

(Page 13, Line 255)

Reviewer #2

Thank you very much for your peer review.
