# Supplementary material for: A multicenter prospective study to determine the optimal range of lymph node dissection in pancreatic cancer surgery after neoadjuvant chemotherapy (LYMRIN-Trial): Project study by the Japan Pancreas Society and JON 2302-P
Source: PLoS One. 2025 Jun 17;20(6):e0325667. doi: 10.1371/journal.pone.0325667 (PMC12173190; doi:10.1371/journal.pone.0325667)
Supplement: Attachment — Submitted filename: Response to Reviewers R3.docx [file pone.0325667.s007.docx]

Response to the comments of the editor and the reviewers

Editor

Thank you for reviewing and handling our manuscript entitled " A multicenter prospective study to determine the optimal range of lymph node dissection in pancreatic cancer surgery after neoadjuvant chemotherapy (LYMRIN-Trial): Project study by the Japan Pancreas Society and JON 2302-P". We re-wrote the manuscript and reference according to Journal requirements. The manuscript was brushed up. We have marked in red the changes made in the manuscript.

Journal Requirements:

[Please review your reference list to ensure that it is complete and correct. If you have cited papers that have been retracted, please include the rationale for doing so in the manuscript text, or remove these references and replace them with relevant current references. Any changes to the reference list should be mentioned in the rebuttal letter that accompanies your revised manuscript. If you need to cite a retracted article, indicate the article’s retracted status in the References list and also include a citation and full reference for the retraction notice.]

We reviewed the reference list and made changes to several references.

Citation numbers 2 and 3 were changed to the journal articles because they were citing online sites.

2. Chen C, Yin L, Lu C, Wang G, Li Z, Sun F, et al. Trends in 5-year cancer survival disparities by race and ethnicity in the US between 2002-2006 and 2015-2019. Sci Rep. 2024;14(1):22715. doi: 10.1038/s41598-024-73617-z.

3. Isaji S, Mizuno S, Windsor JA, Bassi C, Fernández-Del Castillo C, Hackert T, et al. International consensus on definition and criteria of borderline resectable pancreatic ductal adenocarcinoma 2017. Pancreatology. 2018;18(1):2-11. doi: 10.1016/j.pan.2017.11.011.

(Page 24)

Citation number 4 was changed because the journal article was published online ahead.

4. Unno M, Motoi F, Matsuyama Y, Satoi S, Toyama H, Matsumoto I, et al. Neoadjuvant chemotherapy with gemcitabine and S-1 versus upfront surgery for resectable pancreatic cancer: Results of the randomized phase II/III Prep-02/JSAP05 trial. Ann surg. 2025; online ahead of print. doi: 10.1097/SLA.0000000000006730.

(Page 24)

Citation 12 was removed because it was non-public data using the National clinical database.

Reviewer #1

Thank you very much for your peer review.
